# Supplementary material for: Genetic Diversity of Actinobacillus pleuropneumoniae Serovars in Hungary
Source: Vet Sci. 2022 Sep 20;9(10):511. doi: 10.3390/vetsci9100511 (PMC9607985; doi:10.3390/vetsci9100511)
Supplement: Supplementary file 1 [file vetsci-09-00511-s001.zip › Supplementary Table S1.pdf]

Supplementary Table S1.: Characteristics of isolates. ND: not determined; empty cells in columns settlement of origin and year mark missing data

isolate	pulsotype	biotype	serovariant	organ	settlement of origin	year	resistance gene	toxin gene profile
A164	A1	2	13	lung		2008	-	apxIB, apxII, apxIV
A185	A1	2	13	lung	Dalmand	2009	tetL	apxIB, apxII, apxIV
A143	A1	2	13	lung	Dalmand	2006	tetL	apxIB, apxII, apxIV
A138	A1	2	13	lung	Töltéstava	2006	-	apxIB, apxII, apxIV
A142	A1	2	13	lung	Dalmand	2006	tetL	apxIB, apxII, apxIV
A13	A1	2	13	lung		1989	-	apxIV
A131	A1	2	13	lung	Kisújszállás	2006	-	apxIB, apxII, apxIV
A125	A1	2	13	lung	Dalmand	2006	tetL	apxIB, apxII, apxIV
A162	A1	2	13	lung		2008	tetB	apxIB, apxII, apxIV
A163	A1	2	13	lung		2008	-	apxIB, apxII, apxIV
A165	A1	2	13	lung		2008	-	apxIB, apxII, apxIV
211/13	A1	2	13	lung	Ormándlak	2013	-	apxIB, apxII, apxIV
51/95	A1	2	13	lung		1995	-	apxIB, apxII, apxIV
A184	A1	2	13	lung	Dalmand	2009	tetL	apxIB, apxII, apxIV
A150	A2	2	13	lung	Bábolna	2006	tetL	apxIB, apxII, apxIV
A11	A2	2	13	lung			tetL	apxIB, apxII, apxIV
A144	A2	2	13	lung		2006	-	apxIB, apxII, apxIV
A137	A2	2	13	lung		2005	-	apxIB, apxII, apxIV
A145	A2	2	13	lung		2005	-	apxIB, apxII, apxIV
A141	A2	2	13	lung		2006	-	apxIB, apxII, apxIV
A140	A2	2	13	lung	Gerjen	2006	tetL, blaROB	apxIB, apxII, apxIV
A134	A2	2	13	lung	Baja	2006	-	apxIB, apxII, apxIV
A183	A2	2	13	lung	Gerjen	2009	tetL	apxIB, apxII, apxIV
A16	A2	2	13	lung		1998	-	apxIB, apxII, apxIV
84/14	A2	2	13	endocardium		2014	tetL	apxIB, apxII, apxIV
A149	B	2	14	lung		2005	-	apxIB, apxII, apxIV
A12	B	2	14	lung	Dalmand		-	apxIB, apxII, apxIV
A148	U	2	ND	lung	Hercegszántó	2005	-	apxIV
A146	C	1	9	lung	Szeghalom	2003	-	apxIA, apxIB, apxII, apxIV
A129	C	1	9	lung		2006	-	apxIA, apxIB, apxII, apxIV
A147	C	1	9	lung	Szeghalom	2003	-	apxIA, apxIB, apxII, apxIV
A174	C	1	9	lung			-	apxIA, apxIB, apxII, apxIV

A175	C	1	9	lung			-	apxIA, apxIB, apxII, apxIV
A170	C	1	9	lung		2009	-	apxIA, apxIB, apxII, apxIV
8/12	C	1	9	lung	Barcs	2012	-	apxIA, apxIB, apxII, apxIV
327/12	C	1	9	lung	Marcaltő	2012	-	apxIA, apxIB, apxII, apxIV
16/14	C	1	9	lung	Környe	2014	-	apxIA, apxIB, apxII, apxIV
A169	C	1	9	lung		2009	-	apxIA, apxIB, apxII, apxIV
A31	C	1	9	lung	Pötréte	2002	tetH	apxIA, apxIB, apxII, apxIV
112/14	U	2	ND	lung	Püspökladány	2014	tetB	apxIB, apxII, apxIV
206/14	U	2	ND	lung	Bajánsenye	2014	-	ND
32/14	U	2	ND	lung	Fábiánsebestyén	2014	-	ND
270/12	D1	1	16	lung	Komárom	2012	tetB	apxIA, apxIB, apxII, apxIV
272/12	D1	1	16	lung	Csém	2012	tetL	apxIA, apxIB, apxII, apxIV
51/14	D1	1	16	lung	Hajdúszoboszló	2014	-	apxIA, apxIB, apxII, apxIV
85/14	D1	1	16	lung	Karcag	2014	-	apxIA, apxIB, apxII, apxIV
107/14	D1	1	16	lung	Hajdúnánás-Tedej	2014	-	apxIA, apxIB, apxII, apxIV
205/14	D1	1	16	lung	Hajdúszoboszló	2014	-	apxIA, apxIB, apxII, apxIV
A78	D2	1	10	lung		2003	-	ND
A89	D2	1	10	lung			-	ND
A44	D2	1	10	lung		2002	-	ND
A79	D2	1	10	lung		2003	-	ND
145/14	D2	1	10	lung	Bajánsenye	2014	-	apxIA, apxIB, apxII, apxIV
286/12	E	1	untypeable	lung	Elek	2012	-	apxIA, apxIB, apxII, apxIV
203/14	E	1	untypeable	lung	Elek	2014	-	apxIA, apxIB, apxII, apxIV
A7	U	1	12	lung		1996	-	ND
287/12	U	1	12	lung	Komárom	2012	-	apxIB, apxII, apxIV
A168	U	1	ND	lung	reference strain		-	ND
A173	U	1	1/5b	lung			-	ND
A96	U	1	ND	lung			-	ND
A172	U	1	1/5a	lung			-	ND
A123	U	1	ND	lung		2005	-	ND
A72	F	1	ND	lung	Pápa	2003	tetB	ND
A74	F	1	ND	lung	Pápa	2003	tetB	ND
322/12	G1	1	2	lung	Nagyhegyes	2012	-	apxIB, apxII, apxIII, apxIV
67/14	G1	1	2	lung	Hajdúdorog	2014	-	apxIB, apxII, apxIII, apxIV
A187	G2	1	2	lung		2009	-	apxIB, apxII, apxIII, apxIV

309/12	G2	1	2	lung	Felsőcikola	2012	-	apxIB, apxII, apxIII, apxIV
323/12	G2	1	2	lung	Nagyhegyes	2012	-	apxIB, apxII, apxIII, apxIV
A151	G3	1	2	lung	Városföld	2006	-	apxIB, apxII, apxIII, apxIV
A126	G3	1	2	lung		2006	-	apxIB, apxII, apxIII, apxIV
A40	H	1	2	lung		2002	-	apxIB, apxII, apxIII, apxIV
A63	H	1	2	lung		2002	-	apxIB, apxII, apxIII, apxIV
329/12	H	1	2	lung	Marcaltő	2012	-	apxIB, apxII, apxIII, apxIV
A37	H	1	2	lung		2001	-	apxIB, apxII, apxIII, apxIV
A71	H	1	2	lung		2001	-	apxIB, apxII, apxIII, apxIV
262/12	I1	1	2	lung	Rábaszentandrás	2012	tetL	apxIB, apxII, apxIII, apxIV
276/12	I1	1	2	lung	Beremend	2012	tetL	apxIB, apxII, apxIII, apxIV
346/12	I1	1	2	lung	Tiszatenyő	2012	-	apxIB, apxII, apxIII, apxIV
A132	I1	1	2	lung	Sárbogárd	2006	-	apxIB, apxII, apxIII, apxIV
A76	I1	1	2	lung	Nagyigmánd	2003	-	apxIB, apxII, apxIII, apxIV
A130	I1	1	2	lung	Besenyő	2006	-	apxIB, apxII, apxIII, apxIV
86/14	I2	1	2	lung	Derecske	2014	-	apxIB, apxII, apxIII, apxIV
198/14	I2	1	2	lung	Nagyigmánd	2014	-	apxIB, apxII, apxIII, apxIV
202/14	I2	1	2	lung	Küngös	2014	-	apxIB, apxII, apxIII, apxIV
246/12	I2	1	2	lung	Gyömöre	2012	tetL	apxIB, apxII, apxIII, apxIV
320/13	I2	1	2	lung	Hódmezővásárhely	2013	-	apxIB, apxII, apxIII, apxIV
200/14	I2	1	2	lung	Lébény	2014	-	apxIB, apxII, apxIII, apxIV
209/14	I2	1	2	lung	Csillagpuszta	2014	-	apxIB, apxII, apxIII, apxIV
A48	I2	1	2	lung	Pásztó	2002	-	apxIB, apxII, apxIII, apxIV
A45	I2	1	2	lung	Lajoskomárom	2002	-	apxIB, apxII, apxIII, apxIV
A41	I2	1	2	lung		2002	-	apxIB, apxII, apxIII, apxIV
A133	I2	1	2	lung	Pásztó	2006	-	apxIB, apxII, apxIII, apxIV
A136	I2	1	2	lung	Pásztó	2006	-	apxIB, apxII, apxIII, apxIV
A122	I2	1	2	lung	Pásztó	2004	-	apxIB, apxII, apxIII, apxIV
A127	I2	1	2	lung	Pásztó	2006	-	apxIB, apxII, apxIII, apxIV
A42	I2	1	2	lung	Pásztó	2002	-	apxIB, apxII, apxIII, apxIV
A109	I2	1	2	lung		2002	-	apxIB, apxII, apxIII, apxIV
A27	I2	1	2	lung		2002	-	apxIB, apxII, apxIII, apxIV
A167	I2	1	2	lung	Kerékteleki	2008	-	apxIB, apxII, apxIII, apxIV
A166	I2	1	2	lung	Kerékteleki	2008	-	apxIB, apxII, apxIII, apxIV
A186	I2	1	2	lung	Pirító	2009	-	apxIB, apxII, apxIII, apxIV

A182	I2	1	2	lung	Piritó	2009	-	apxIB, apxII, apxIII, apxIV
A181	I2	1	2	lung	Pásztó	2009	-	apxIB, apxII, apxIII, apxIV
249/12	I2	1	2	lung	Mocsa	2012	-	apxIB, apxII, apxIII, apxIV
208/14	I2	1	2	lung	Tárkány	2014	-	apxIB, apxII, apxIII, apxIV
196/12	I2	1	2	lung	Küngös	2012	tetB	apxIB, apxII, apxIII, apxIV
26/14	I2	1	2	lung	Hajdúböszörmény	2014	-	apxIB, apxII, apxIII, apxIV
A19	I2	1	2	lung		1998	-	apxIB, apxII, apxIII, apxIV
416/13	I3	1	2	lung	Tab	2013	-	apxIB, apxII, apxIII, apxIV
A171	I3	1	2	lung			-	apxIB, apxII, apxIII, apxIV
A68	J	1	2	lung	Bábolna	2002	-	apxIB, apxII, apxIV
A70	J	1	2	lung	Bábolna	2002	-	apxIB, apxII, apxIV
A67	J	1	2	lung	Bábolna	2002	-	apxIB, apxII, apxIV
