# Supplementary material for: Genetic Diversity of Actinobacillus pleuropneumoniae Serovars in Hungary
Source: Vet Sci. 2022 Sep 20;9(10):511. doi: 10.3390/vetsci9100511 (PMC9607985; doi:10.3390/vetsci9100511)
Supplement: Supplementary file 1 [file vetsci-09-00511-s001.zip › Supplementary Table S2.pdf]

Supplementary Table S2.: Genome contiguity statistics as output by quast.

Assembly	51/95	A12	A149	A16	84/14	A125	A140	A185	211/13
# contigs (>= 0 bp)	146	99	182	182	2673	104	137	97	1713
# contigs (>= 1000 bp)	44	41	51	51	98	49	49	48	84
# contigs (>= 5000 bp)	32	33	37	37	60	38	36	39	58
# contigs (>= 10000 bp)	29	27	28	28	53	32	33	34	51
# contigs (>= 25000 bp)	25	24	25	25	32	26	27	27	34
# contigs (>= 50000 bp)	11	13	12	12	12	12	11	11	13
Total length (>= 0 bp)	2282301	2276316	2329525	2329525	3012367	2266880	2274712	2266725	2724300
Total length (>= 1000 bp)	2254886	2260285	2293572	2293572	2300554	2257902	2255931	2258183	2273306
Total length (>= 5000 bp)	2230146	2242443	2258528	2258528	2222940	2230617	2227165	2237250	2224147
Total length (>= 10000 bp)	2209874	2201550	2197563	2197563	2175036	2190547	2204787	2204585	2177961
Total length (>= 25000 bp)	2150242	2156084	2156307	2156307	1838952	2102072	2117766	2100483	1919546
Total length (>= 50000 bp)	1621739	1732583	1636012	1636012	1102269	1574432	1523396	1468601	1174745
# contigs	129	79	158	158	2530	58	78	57	1621
Largest contig	331517	331330	331464	331464	157667	296759	296311	246581	175883
Total length	2280089	2273812	2326649	2326649	2993898	2262334	2268791	2262903	2711841
GC (%)	41.21	41.14	41.15	41.15	41.03	41.16	41.18	41.17	41.26
N50	132709	121850	111799	111799	35966	108593	108593	109394	43028
N75	49412	53844	48924	48924	4229	47431	47280	47380	16717
L50	6	7	7	7	21	7	7	7	17
L75	13	13	15	15	65	15	15	16	40
# N's per 100 kbp	51.93	17.15	16.76	16.76	13.03	19.18	17.32	21.39	14.60
